# Concern about Workplace Violence and Its Risk Factors in Chinese Township Hospitals: A Cross-Sectional Study

**DOI:** 10.3390/ijerph13080811

**Published:** 2016-08-10

**Authors:** Kai Xing, Xue Zhang, Mingli Jiao, Yu Cui, Yan Lu, Jinghua Liu, Jingjing Zhang, Yuchong Zhao, Yanming Zhao, Ye Li, Libo Liang, Zheng Kang, Qunhong Wu, Mei Yin

**Affiliations:** 1 Department of Health, Policy and Hospital Management, School of Public Health, Harbin Medical University, Harbin 150081, China; xingkai19921202@163.com (K.X.); Liye19832014@126.com (Y.L.); 2 School of Humanities and Social Sciences, Harbin Medical University, Harbin 150081, China; xuezhang1212@sina.com; 3 Institute of Quantitative and Technical Economics, Chinese Academy of Social Science, Beijing 100000, China; 4 Department of Social Medicine, School of Public Health, Harbin Medical University, Harbin 150081, China; yucuicuicui@126.com (Y.C.); lianglibo2015@163.com (L.L.); zhengkang1983@126.com (Z.K.); 5 School of Public Health, Jiamusi University, Jiamusi 154007, China; jmsly@163.com; 6 School of Public Health, QiQihar Medical University, QiQihar 161006, China; jinghualiu1@sina.com; 7 Continuing Education Section, Third Affiliated Hospital of QiQihar Medical University, QiQihar 161000, China; jingjing_she@126.com; 8 Department of Publicity and United Front Work, Heilongjiang Nursing College, Harbin 150081, China; zhaoyuchong@foxmail.com; 9 Department of Computed Tomography, The 2nd Affiliated Hospital of Harbin Medical University, Harbin 150081, China; yanmingzhao1@sina.com

**Keywords:** worry, risk factors, township hospitals, workplace violence

## Abstract

Workplace violence in Chinese township hospitals is a major public health problem. We identified the risk factors of healthcare workers’ worry about experiencing workplace violence in 90 Chinese township hospitals and determined specific measures for differing stages of violence (based on crisis management theory). Participants were 440 general practitioners and 398 general nurses from Heilongjiang Province, China (response rate 84.6%). One hundred and six (12.6%) respondents reported being physically attacked in their workplace in the previous 12 months. Regarding psychological violence, the most common type reported was verbal abuse (46.0%). While most (85.2%) respondents had some degree of worry about suffering violence, 22.1% were worried or very worried. Ordinal regression analysis revealed that being ≤35 years of age, having a lower educational level, having less work experience, and working night shifts were all associated with worry about workplace violence. Furthermore, those without experience of such violence were more likely to worry about it. Respondents’ suggested measures for controlling violence included “widening channels on medical dispute solutions,” “improving doctor-patient communication,” and “advocating for respect for medical workers via the media.” Results suggest the target factors for reducing healthcare workers’ worry by according to the type of education and training and possible measures for limiting workplace violence in township hospitals.

## 1. Introduction

In recent years, workplace violence (WPV) against healthcare workers has become a worldwide problem [1]. Violence in the healthcare sector can have serious personal consequences for healthcare workers, such as a loss of consciousness, need for medical treatment, disability, and even death, lost workdays and absenteeism, increased occupational strain, employment termination, or turnover [2]. In addition, WPV has serious consequences for both patients and the facilities because the perceived likelihood of violence is related to adverse patient outcomes due to the poorer quality of care and treatment provided [3]. Furthermore, consequences such as reduced work performance, motivation and morale, and creativity can crucially influence the effectiveness of health systems as a whole, especially those in developing countries [4,5].

Recently, WPV directed at healthcare workers, especially doctors, is becoming increasingly prevalent, thus making medical practice in China a high-risk job. Since 2000, there has been an 11% annual increase in physical violence incidents perpetrated by patients against doctors. The prevalence of such violence was 8.3% in 2012, almost twice that of 2008 (4.5%) [6]. The number of extreme cases is also increasing: in 2012 alone, seven medical workers were killed by patients, or approximately half of the total number of similar deaths in the previous nine years [7]. Given this background, increasingly more healthcare workers are worrying about their own personal safety.

WPV is increasingly being recognized as a serious problem in general practice and public health. WPV is common in countries with comparable primary medical care systems: New Zealand, the United Kingdom, and Australia [8,9,10,11]. Health professionals in the UK, especially general practitioners (GPs), are consistently at risk of WPV [12], with around 10%–11% of GPs reportedly experiencing assault, 5% threats with a weapon, and 25%–59% verbal abuse [13,14,15]. Additionally, 63.7% of sampled Australian GPs had experienced some violence at work [16]. Research has identified a number of risk factors of WPV. For instance, Magin et al. reported that GPs who are female and who have fewer years of experience have a higher likelihood of violence [16]. In addition, nursing is also a high-risk occupation for violent incidents in many countries. A national study found 80% of Canadian nurses reported some form of violence during their career [17]. In Switzerland, 72% of nurses reportedly experienced verbal violence and 42% physical violence in the past 12 months [18]. In Australia, the majority (89.5%) of nurses defined aggressive behavior as including verbal and physical aggression and intimidation [19]. Many researchers have also identified a number of risk factors of WPV among nurses. For example, a study in China revealed that age, years of experience, working in shifts, anxiety level and level of education are associated with WPV among nurses [20].

In China, township hospitals are the hubs of the rural tertiary health service system, dealing primarily with common disease management. In such hospitals, the status and job characteristics of GPs have made them the focus of future medical team construction [21]. However, most township hospitals currently lack a sufficient number of GPs, making it difficult for them to meet the care needs of villagers [21]. According to a Ministry of Health report [22], there were over 37,097 township hospitals across China in 2013, of which 996 were in Heilongjiang Province. Within these 996 hospitals, there were 2081 GPs and 3616 registered nurses.

Worry is a form of perseverative cognition characterized by recurrent thoughts about potential negative events and attempts to mentally solve these future problems [23]. It is closely related to anxiety, being a common feature and diagnostic criterion of many anxiety disorders [24]. Anxiety is an unpleasant psychological and physiological state with cognitive, somatic, emotional, and behavioral components, characterized by feelings of uneasiness, apprehension, fear, or worry [25]. Anxiety is one of the most common psychiatric conditions encountered in primary care [26,27]. While a normal reaction to stress or threat, and helpful for dealing with stressful or threatening situations, anxiety can become a disabling medical condition when excessive or persistent, and can worsen if left untreated [25].

Healthcare workers have a high risk of developing anxiety, which can impair their quality of life, influence their job performance, affect patient safety, and lead to changes in immune system functioning [28,29,30]. Healthcare workers’ anxiety undoubtedly has profound social effects, so more attention should be directed toward reducing anxiety and worry, especially about WPV.

There are a variety of measures for dealing with WPV among healthcare workers. To investigate how it is currently handled, we applied crisis management theory. Crisis management refers to the discipline of managing major threats that may harm an organization, its various stakeholders, or the general population. It specifically involves managing these events (or crises) before, during, and after they have occurred. Lerbinger [31] proposed that there are eight types of crises, of which one is WPV.

Overall, numerous studies have been conducted on WPV and worry. However, no consensus has been reached on the state of healthcare workers’ worry about WPV in township hospitals in China or the risk factors. As such, we adopted an exploratory approach to this topic. Specifically, we identified the prevalence and level of severity of physical and psychological violence against GPs and nurses in township hospitals in Heilongjiang Province in northeastern China, and the risk factors contributing to healthcare workers’ worry. Furthermore, drawing on crisis management theory, we identified several measures of coping with WPV and determined respondents’ perceptions of them. 

## 2. Materials and Methods

A cross-sectional study using a questionnaire survey was conducted in Heilongjiang Province, China. In 2012, Heilongjiang had a population of 38.1 million and a total of 996 township hospitals. We administered the survey in three locations: Harbin, Daqing, and Hegang. Because of the study’s time and resource limitations, we purposively selected 90 township hospitals (approximately 10%) according to the geographical location, populations, and health status of the townships in which they were located. This comprised 30 hospitals in Harbin (in the middle of the province), 30 hospitals in Daqing (in the west), and 30 hospitals in Hegang (in the east). Permission to administer the survey was obtained from all 90 township hospitals. The collected data were used in a previous article published in 2015 [32], however, because of the different dependent variable used in that case, the number of valid questionnaires in this study decreased slightly, from 840 to 838. 

### 2.1. Data Collection

The survey was conducted from September to November 2014. Access to participants was negotiated through supervisors in every study hospital. We distributed an anonymous, self-administered questionnaire to each participant, along with a notification letter and return envelope. A box was kept in the department manager’s office into which participants could put completed and sealed questionnaires to ensure privacy and anonymity. The study purpose and rights of the healthcare workers regarding participation were declared in the letter. Participants had 7 days to complete the questionnaire. All of the returned questionnaires were secured in a locked room that could only be accessed by research personnel. A total of 990 questionnaires were distributed, of which 838 valid questionnaires were obtained (response rate = 84.6%).

### 2.2. Questionnaire

The questionnaire used was developed according to a literature review and a modified version of the questionnaire developed in 2003 by a joint program of the International Labour Office (ILO), International Council of Nurses, WHO, and Public Services International [33]. First, we formally obtained documented permission to use the questionnaire from the ILO and WHO. It was then translated into Mandarin Chinese and back-translated into English to verify the accuracy of the Mandarin version. Subsequently, the questionnaire was modified to fit our study objectives and the township hospital context in China. For example, Yi Nao was considered a form of psychological violence due to the particularity of WPV in China. Hesketh and Wu described Yi Nao as gangs consisting “largely of unemployed people with a designated leader. They threaten and assault hospital personnel, damage facilities and equipment, and prevent the normal activities of the hospital” [20]. The content validity was determined by 18 healthcare-related experts throughout China, who were asked to assess the questionnaire in terms of clarity, relevance, comprehensiveness, and sensitivity to China’s culture. Upon revision by the expert committee, we administered the questionnaire to 30 participants as a pre-test (all of them were subsequently excluded from the study). As per these individuals’ feedback, further modifications were undertaken. For all questions, the Cronbach’s alpha coefficient was 0.86. 

The modified questionnaire has four sections: (1) respondents’ demographic characteristics and workplace data; (2) questions regarding physical violence, including its prevalence, perpetrators’ demographic characteristics, time of attack, tools used in the attack, consequences for the perpetrators, etc.; (3) questions regarding mental and emotional violence; and (4) organizational measures, including incident reporting, supervisor support, training programs, etc. In the second and third sections, we included the definitions of violence that we used, which were derived from the WHO [34]. Physical violence refers to the use of “physical force against another person or group, that results in physical, sexual or psychological harm.” It can include beating, kicking, slapping, stabbing, shooting, pushing, biting, and pinching, among others. In contrast, psychological violence is defined as “intentional use of power, including threat of physical force, against another person or group, that can result in harm to physical, mental, spiritual, moral, or social development.” It includes verbal abuse (e.g., humiliating or degrading behavior, lack of respect), Yi Nao, threats (e.g., offensive behavior or comments that represent threats or elicit fear), verbal sexual harassment, and physical sexual harassment (e.g., unwelcome behavior or comments of a sexual nature). In the organizational measures section, drawing on crisis management theory, we grouped the various measures of dealing with WPV into “preventive measures,” “control measures during violence,” and “intervention measures after violence.” Respondents selected the most important and effective measures (multiple could be chosen) according to their own opinions.

In this study, we focused on the frequency of WPV and the degree of worry about it. To assess the former variable, the following questions were used: “In the last 12 months, have you been physically attacked in your workplace?” and “In the last 12 months, have you suffered psychological violence in your workplace?” Regarding the latter variable, a five-point scale ranging from “absolutely not worried” to “very worried” was used in response to the question: “How worried are you about violence in your current workplace?”

### 2.3. Data Analysis

After coding the data with EpiData, we asked another member of our team to verify the accuracy of the entered data. The statistical analyses were conducted with IBM SPSS Statistics 19.0 (IBM Corp., Armonk, NY, USA). All of the analyses began with basic descriptive statistics of the demographic characteristics, the frequency of WPV, and healthcare workers’ degree of worry about suffering from WPV. Then, ordinal regression analysis was used to determine the potential associations between worry about suffering WPV and respondents’ characteristics, including age, gender, years of experience in the current workplace, education level, occupation, professional title, amount of direct physical contact (e.g., washing, turning, lifting) with patients, whether they worked night shifts, whether they had experienced various types of WPV, and whether they had had training to manage aggressive or violent situations. The gradual filtering method of logistic regression was used, wherein variable selection involved entering the independent variables into the ordinal regression model and excluding them according to a *p* < 0.05. Age, years of experience, educational level, night shift, and experience of physical violence and *Yi Nao* were included in the final ordinal regression model. Odds ratios (OR) and 95% confidence intervals (CI) were calculated; *p* < 0.05 was considered statistically significant.

### 2.4. Ethical Approval

Ethical approval was granted by the Institutional Review Board of Harbin Medical University before data collection commenced (Project Identification Code: HMUIRB20160014). All of the study procedures were approved by each study hospital, and all of the participants gave their informed consent to participate.

## 3. Results

Among the 838 questionnaires returned, 440 were from GPs and 398 were from general nurses. Both the descriptive and multiple regression analysis results are presented below.

### 3.1. Respondents’ Demographic Characteristics

A summary of these characteristics are shown in Table 1.

### 3.2. Prevalence of Workplace Violence 

Table 2 shows that the prevalence of physical violence experienced in the previous 12 months was 12.6% (*n* = 106). Regarding psychological violence, the most common type was verbal abuse (33.9%), followed by Yi Nao (17.0%) and threats (16.5%).

### 3.3. Degree of Worry about Suffering Workplace Violence 

Table 3 suggests that the majority of respondents (85.2%; *n* = 716) had some degree of worry about suffering violence in their workplace. However, 22.1% (*n* = 186) reported being either worried or very worried about suffering violence.

### 3.4. Intervention Strategies against Workplace Violence

As shown in Table 4, in terms of preventive measures, most respondents chose “widen the channels of medical dispute resolution” (61.6%), and “make fees more transparent” (59.9%). For the control measures, 81.0% of the respondents believed it necessary to “improve doctor-patient communication skills.” Finally, for intervention measures, “advocate for respect of medical workers via the media” was thought to be an effective measure for minimizing losses due to violence.

### 3.5. Ordinal Regression Analysis

The ordinal logistic regression analysis revealed that age, years of experience, educational level, working in a night shift, and having previous experience with physical violence, Yi Nao, and sexual harassment were all significantly associated with worry about WPV in Table 5. Specifically, individuals who were 35 years old or older had lower odds of worrying about WPV. In contrast, those with a lower educational level and fewer years of experience had greater odds of worrying about WPV. Working the night shift was associated with greater odds of worrying, as was having experience of physical violence, Yi Nao, and sexual harassment.

## 4. Discussion

We found that 85.2% of respondents had some degree of worry about WPV, and 22.1% were either worried or very worried. These results confirm that healthcare workers’ worry about violence is a serious problem requiring immediate attention from policymakers and hospital managers. Until now, there have been no related articles on the relationship between healthcare workers’ worry and WPV, although some previous researchers have explored the relationship between anxiety and WPV [35,36].

Previous research has indicated that age is a risk factor for WPV in Chinese township hospitals, with respondents who are younger having an increased likelihood of experiencing violence [32]. Similarly, our findings indicated that respondents who were 35 years or younger had a higher odds of worrying about suffering violence in township hospitals. 

Previous researchers have also found that medical staff with lower educational levels are more likely to suffer from WPV [37,38]; thus, it is unsurprising that those with lower educational levels in our study were more likely to worry about being subjected to WPV. Such healthcare workers might be more likely to worry about violence because they lack sufficient professional and patient-doctor communication skills. 

Some previous studies indicated that the majority of WPV occurred during the night shift [37,39,40]. Similarly, working a night shift was found to be a risk factor for increased worry about suffering violence in our own study. Working the night shift is usually associated with working alone for long hours, which is similarly considered a risk factor for WPV. Additionally, working during the night increases the risk of exposure to WPV due to lax security [41,42]. It may help to limit the opportunities for workers to be alone and engage in after-hours care to reduce the risk of WPV [41,43]. These measures may be similarly effective in reducing healthcare workers’ worry about WPV.

The most important finding in our study was that individuals who had previous experience of physical violence and Yi Nao were more likely to worry about suffering WPV. Similarly, previous researchers have found that healthcare workers’ exposure to WPV is related to a high degree of anxiety state [20,44]. Such participants should be provided with psychological counseling services and targeted training against violence.

Regarding respondents’ attitudes towards the measures for violence prevention and intervention, the most commonly cited measures were “widening the channels of medical dispute resolution” and “advocate for respect of medical workers via the media,” which are associated with the current situation in the Chinese medical industry. In China, it is not an uncommon phenomenon that some hospitals engage in unreasonable treatment of patients during medical disputes in order to protect their own reputation. These solutions are often conducted in private and without formal proceedings, which can increase the likelihood of violence perpetrated by patients and their families [45]. In addition, a major factor stimulating WPV against healthcare workers is shallow or erroneous reports of medical disputes by new media. A study on healthcare workers in Guangzhou, China indicated that around 79.6% of medical staff perceived the negative coverage by the media as the original trigger of many WPV incidents [46]. To solve this problem, media reports should engage in a deeper or more thorough analysis of events from a neutral point of view and try to make more impartial judgments based on fact. Additionally, hospitals should quickly and positively respond to medical disputes, and eliminate negative influence as soon as possible. For example, hospitals can make emergency plans, strengthen crisis-monitoring efforts and expand their own social influence through positive advertising. At the government level, the government should increase penalties for shallow or erroneous reports of medical disputes by new media. Finally, medical staff might learn to use legal weapons to protect their own rights and interests.

### Limitations

This study has several limitations. First, because of the time and resource restrictions, our study was limited to only one province in China and 90 purposively selected township hospitals; combined with the relatively small sample size, we cannot generalize our findings to all township hospitals in Heilongjiang province or across China. A larger survey in more than one province is needed to provide a more accurate description of WPV in other township hospitals. Nevertheless, our findings can offer a guide for such research. Second, we used a self-administered questionnaire to collect data, which may have led to recall bias among respondents. Finally, our study used only a single item rather than a validated psychological scale to measure healthcare workers’ worry about WPV. Nevertheless, our main purpose was not to determine healthcare workers’ levels of worry, but to clarify how they might be more likely to suffer from WPV through understanding their worry.

## 5. Conclusions

We identified the risk factors of healthcare workers’ worry about WPV in Chinese township hospitals, which can help us identify those who are more likely to worry about WPV in daily work, whom we might target with mental health support and anti-violence training to further protect their physical and mental health. Furthermore, we proposed several possible countermeasures for the different stages of WPV, which may help in guiding how to prevent and respond to WPV in Chinese township hospitals.

## Figures and Tables

**Table 1:** Demographic characteristics of the respondents (*N* = 838).

Characteristics	N	%
Gender		
Male	442	52.7
Female	396	47.3
Age		
≤35	140	16.7
35–45	422	50.4
≥45	276	32.9
Years of experience		
1–10	116	13.9
11–20	390	46.5
>20	332	39.6
Educational level		
Postgraduate	8	1.0
Undergraduate	376	44.9
College	350	41.8
Technical secondary school education or below	104	12.4
Professional title		
Senior	172	20.5
Intermediate	406	48.4
Junior	192	22.9
No title	68	8.1
Occupation		
General practitioner	440	52.5
General nurse	398	47.5

**Table 2:** Prevalence of workplace violence in the past 12 months (*N* = 838).

Type of Violence Type	N	%
Physical violence	106	12.6
Verbal abuse	285	34.0
Yi Nao	143	17.1
Threats	138	16.5
Verbal sexual harassment	102	12.2
Physical sexual harassment	64	7.6

**Table 3:** Prevalence of different degrees of worrying about suffering workplace violence (*N* = 838).

Degree of Worry	N	%
Absolutely not worried	124	14.8
A little worried	352	42.0
Moderately worried	176	21.0
Worried	100	11.9
Very worried	86	10.3

**Table 4:** Strategies for managing workplace violence as suggested by participants (*N* = 838).

Intervention Strategies	Specific Strategies	N	%
Preventive measures before violence	Widen the channels of medical dispute resolution	516	61.6
	Make fees more transparent	502	59.9
	Carry out targeted training to strengthen healthcare workers’ capacity to deal with violence	491	58.6
	Improve treatment and care quality and diagnostic accuracy	478	57.0
	Workplace violence legislation	444	53.0
	Develop violence prevention guidelines or plans	340	40.5
Control measures during violence	Improve doctor-patient communication skills	679	81.0
	Shorten waiting times	502	59.9
Intervention measures after violence	Advocate for respect of medical workers via the mass media	490	58.5
	Improve violence reporting, statistics, and intervention mechanisms	327	39.0

**Table 5:** Ordinal regression results on the risk factors contributing to worry about workplace violence.

Variable	Worry about Workplace Violence		
Age	*p*	Adjusted ORs	95% CIs
≤35		1	
35–45	0.0064	0.38	(0.189, 0.762)
≥45	0.01	0.614	(0.424, 0.89)
Years of experience			
<10	0.0048	2.866	(1.379, 5.959)
10–20	<0.0001	2.234	(1.556, 3.208)
>20		1	
Educational level			
Technical secondary school education or below	0.0127	1.28	(1.054, 1555)
Postgraduate/undergraduate/college		1	
Night shift			
Yes		1	
No	<0.0001	0.372	(0.267, 0.519)
Previous experience of physical violence			
Yes	0.0033	1.861	(1.23, 2.817)
No		1	
Previous experience of Yi Nao			
Yes	<0.0001	1.738	(1.336, 2.261)
No		1	

## Abbreviations

WPV	Workplace violence
WHO	World Health Organization
GPs	General practitioners
